# Mining Association rules for Low-Frequency itemsets

**DOI:** 10.1371/journal.pone.0198066

**Published:** 2018-07-23

**Authors:** Jimmy Ming-Tai Wu, Justin Zhan, Sanket Chobe

**Affiliations:** Department of Computer Science, University of Nevada, Las Vegas, Nevada, United States of America; University of Oregon, UNITED STATES

## Abstract

High utility itemset mining has become an important and critical operation in the Data Mining field. High utility itemset mining generates more profitable itemsets and the association among these itemsets, to make business decisions and strategies. Although, high utility is important, it is not the sole measure to decide efficient business strategies such as discount offers. It is very important to consider the pattern of itemsets based on the frequency as well as utility to predict more profitable itemsets. For example, in a supermarket or restaurant, beverages like champagne or wine might generate high utility (profit), but also sell less frequently compared to other beverages like soda or beer. In previous studies, it is observed that people who buy milk, bread, or diapers from a supermarket, also tend to buy beer or soda. But the items like milk, diapers, beer, or soda generate less utility (profit value) compared to beverages like champagne or wine. If we combine items like champagne or wine having high utility but less frequency, with the frequently sold items like milk, diaper, or beer, we can increase the utility of the transaction by providing some discount offers on champagne or wine. In this paper, we are integrating low-frequency itemsets with high-frequency itemsets, both having low or high utility, and provide different association rules for this combination of itemsets. In this way, we can generate a more accurate measure of pattern mining for various business strategies.

## Introduction

Applications of data mining [1] focus on either generating patterns, or prediction of customer behavior, to generate more profit or decide strategies for the growth of a business. Earlier, market basket analysis focused only on the Frequent Itemset Mining (FIM) [2–7]. Given a transaction database, FIM [2] is used to determine the frequently occurring items in a transaction database, which is considered an important factor in making the business strategies. But the FIM [2] has a limitation that it assigns a similar profit, or weight to all items. For example, consider an electronic retail shop, where, accessories like headphones and chargers are sold frequently, but have low-profit value. The items like laptops and television sets have high-profit value, but, are sold with low-frequency. The FIM gives an equal importance to all the accessories, and rejects high profit items like laptops and television sets due to their low-frequency, which is not ideal for business strategies.

To overcome this limitation, a new approach was discovered known as the High Utility Itemset Mining (HUIM) [8–18]. HUIM [8–18] considers the scenario, where items can appear more than once in a transaction, and have a different weight, or profit value assigned for each item. HUIM [8–18] makes it possible to discover the combination of products with high profit, and help retailers or businesses to build marketing strategies like discount offers to sell these products. Several algorithms like Two Phase [9], EFIM (Efficient high utility Itemset Mining [10–12]), UP-Growth [13], UP-Growth+(Two phase algorithms) [14], and HUI-Miner [16] are already developed to generate the high utility itemsets. However, there is a limitation that these algorithms consider only the utility as a sole measure to generate high utility itemsets. This might result in the generation of itemsets which yield high-profit, but, are weakly correlated [8–18], or have a low-frequency. A novel approach of the combination of the frequent itemset mining with the high utility itemset mining can be introduced to generate more accurate patterns, and derive better business strategies. For example, in supermarkets or restaurants, beverages like champagne, or wine generate high utility, but are sold with less frequency compared to other beverages like beer, or soda. In the previous studies of association rules mining [2], it is observed that whenever customers buy itemsets like milk, bread, or diapers, they also tend to buy beer. Based on the association rule mining, we can sell the combination of low-frequency and high frequency items. Frequently sold items like milk, diapers, or beer which have low utility, can be combined with low-frequency items like champagne, or wine, which have high utility. This combination of low-frequency itemset with high-frequency itemset can generate different association rules, which can be helpful to design effective business strategies. For example, when any customer purchases high-frequency items like milk, diaper, or beer, various discount offers can be provided on low-frequency items like champagne, or wine to attract these customers, and hence increase the sales and overall revenue of the transaction.

This paper focuses on mining the association rules for the combination of low-frequency itemsets having low, or high utility, with the high-frequency itemsets having low, or high utility. The key contributions to the designed algorithm are listed below.

1. Evolutionary work is already done on the frequency and utility mining. We refer this work [2–9, 11–18] to generate the different combination of low-frequency itemsets with the high-frequency itemsets having low, or high utility.
2. In this paper, the traditional measure of association rule mining [2] like *Confidence* and *Support* are used to calculate the association between low-frequency itemsets (low or high utility) and high-frequency itemsets (low or high utility).
3. The experimental results show that the proposed algorithm is able to derive the required association rules to generate more accurate prediction, and business strategies.

The rest of the paper is organized as follows: the section *Related work* focuses on related work, and background. The section *Proposed Approach* describes the proposed algorithm in detail. The section *Experimental Results* shows the experimental results, and the section *Conclusions And Future Work* concludes the work.

## Related work

This section revisits the Association Rule Mining based on the frequency, and high utility itemset mining.

### Apriori algorithm

Since the inception of association rules mining, many algorithms have been developed for the association rule and frequent itemset mining. The Apriori algorithm was first introduced by Agarwal et al. [2] to find the frequent itemsets from a large transaction database. The key concept behind the Apriori algorithm [2] is to eliminate the itemsets with support value less than the *min. support*, subsets of such itemsets are also not frequent itemsets. The support of an itemset never exceeds support of its subsets, this property is known as *Anti-Monotone* property. Consider following example of a transaction database with the frequent itemsets and utility of each item presented in Tables 1 and 2, respectively.

**Table 1:** Transaction database with frequent items.

TID	Transaction	Frequent items
*T*_1_	(A:1), (C:1), (D,1)	(A:3, C:5, D:3)
*T*_2_	(A:2), (C:6), (E:2), (G:5)	(A:3, C:5, E:3)
*T*_3_	(A:1), (B:2), (C:2), (D:6), (E:1), (F:1)	(A:3, B:3, C:5, D:3, E:4)
*T*_4_	(B:4), (C:3), (D:3), (E:1)	(B:3, C:5, D:3, E:4)
*T*_5_	(B:2), (C:2), (E:1), (G:2)	(B:3, C:5, E:3)

**Table 2:** Profit table.

Item	Profit Value
*A*	5
*B*	2
*C*	1
*D*	2
*E*	3
*F*	30
*G*	1

#### Association rules mining

The Apriori algorithm [2] works in multiple phases, where frequent itemsets are determined in each phase from a transaction database. In the first phase, support value of all items are calculated, and frequent items are discovered based on the support value larger than or equal to the minimum threshold support. From the Table 1, we can see that the support value of all the items is as follows: *Sup(A) = 5, Sup(B) = 3, Sup(C) = 5, Sup(D) = 3, Sup(E) = 4, Sup(F) = 1*, and *Sup(G) = 2*. If we consider the minimum support value (*min.Support*) is 3, we can see that *Sup(A), Sup(B), Sup(C), Sup(D)*, and *Sup(E)* ≥ *min.Support*, and hence, the items *A, B, C, D*, and *E* can be considered as the frequent itemsets. In the subsequent phases, individual items are joined together to generate the candidate itemsets, which have the minimum support. Once all the candidate itemsets are generated having support value greater than or equal to *min.Support*, we can determine the association rules for these candidate itemsets, based on the confidence measure. The candidate itemsets are generated by joining items in the same transaction. The confidence measure is used to generate the association rule for the candidate itemsets. The confidence measure takes into account the support value of final itemset, and the support value of the itemset from which the final itemset is derived in the same transaction, also known as an underlying itemset. The confidence measure is defined as the conditional probability of the support value of the final itemset to an underlying itemset. It can also be defined as, the support value of the final itemset divided by the support value of an underlying itemset. If the confidence value of a given association rule is greater than or equal to the *min.Confidence*, that association rule can be used for identifying the frequent itemsets. From the transaction database in Table 1, the candidate itemsets *(A,C), (B,C), (B,E), (C,D), (C,E),* and *(B,C,E)* can be generated with the support value larger than or equal to 3. We can generate the association rules like *C* → (*C*, *E*), or *B* → (*B*, *C*) based on the support, and confidence values. Traditional association rule mining generate a large number of candidate itemsets for a large transaction database. Since the inception of the Apriori algorithm [2], a number of algorithms are developed to optimize the Apriori algorithm [2]. The Apriori algorithm [2] requires several database scans for a large transaction database, and hence, more time to generate the frequent itemsets. Many different tree structures are developed like *FP-tree*, and the pattern growth algorithms like FP-Growth [3] etc., to avoid candidate itemset generation. We focus on mining association rules using the FP-Growth algorithm [3] for our problem.

### High utility mining

The *Frequent Itemset Mining* has an important limitation that it considers each item has a similar utility, or weight value, and gives equal importance to every item in a transaction. To address this limitation, the *Utility Itemset Mining* [8–26] was introduced. The utility mining considers the case where, every item appears more than once and has some weight, or unit profit value assigned to it. The itemsets with utility value greater than or equal to some threshold value are generated, and known as the *High Utility Itemsets*.

#### Utility of an item and itemset

The utility of an item *i*_*j*_ ∈ *T_*c*_* in a transaction database is denoted by *u*(*i*_*j*_, *T*_*c*_), and defined as,

$$\begin{array}{c} u\left(i_{j},T_{c}\right)=q\left(i_{j},T_{c}\right)\times p\left(i_{j}\right) \end{array}$$ (1)

Similarly, the utility of an itemset *X* in a transaction is denoted by *u*(*X*, *T*_*c*_), and defined as,

$$\begin{array}{c} u\left(X,T_{c}\right)=\sum_{i_{j}\subseteq X\cap X\subseteq T_{c}}u\left(i_{j},T_{c}\right) \end{array}$$ (2)

#### Utility of transaction

The transaction utility of a transaction *T*_*c*_ is denoted by *TU*(*T*_*c*_), and defined as,

$$\begin{array}{c} TU\left(T_{c}\right)=\sum_{X\subseteq T_{c}}u\left(X,T_{c}\right) \end{array}$$ (3)

The total utility denoted by *TU* in a database *D* is defined as,

$$\begin{array}{c} TU=\sum_{T_{c}\in D}TU\left(T_{c}\right) \end{array}$$ (4)

From our earlier example, Table 2 shows the utility value (unit profit) for each item in a transaction database. We can calculate the utility of each item as follows, the utility of an item *A* in *T_2_* is *u(A, T_2_)* = *5* × *2* = *10*. The utility of itemset *(A, C)* in T_2_ is *u((A, C), T_2_)* = *u(A, T_2_)* + *u(C, T_2_)* = *5* × *2 + 1* × *6 = 16*. Similarly, the utility of itemset *(A, C)* in every transaction can be calculated, and known as the Utility of itemset in a transaction database, *u((A, C)* = *u((A, C), T_1_)* + *u((A, C), T_2_)* + *u((A, C), T_3_)* = *u(A, T_1_)* + *u(C, T_1_)* + *u(A, T_2_)* + *u(C, T_2_)* + *u(A, T_3_)* + *u(C, T_3_)* = *5* × *1 + 1 + 5* × *2 + 1* × *6 + 5* × *1 + 1 = 28*.

#### High utility itemset

An itemset *X* in a transaction database *D* is a high utility itemset (*HUI*), if its utility is greater than or equal to the user specified minimum threshold, where minimum threshold is specified as *min*.*util*,

$$\begin{array}{c} HUI\leftarrow \{X|u(X)\geq min.util\} \end{array}$$ (5)

If the *min.util* = *30*, we can calculate the high utility itemsets form transaction database shown in (Tables 1 and 2) as follows, *u(B,C) = 30, u(A,C,E) = 31, u(B,C,D) = 34, u(B,C,E) = 31, u(B,D,E) = 36, u(B,C,D,E) = 40,u(A,F) = 30, u(B,F) = 35, u(C,F) = 34, u(D,F) = 31, u(E,F) = 42, u(A,B,F) = 33, u(A,C,F) = 39, u(A,D,F) = 36, u(A,E,F) = 47, u(A,B,C,F) = 41, u(A,B,D,F) = 51, u(A,B,E,F) = 42, u(A,C,D,F) = 49, u(A,C,E,F) = 40, u(A,D,E,F) = 50, and u(A,B,C,D,E,F) = 56* are high utility itemsets.

#### Transaction weighted utilization

The *High Utility Mining* uses an important property known as *transaction weighted utilization*, for pruning the search space. The *transaction weighted utilization (TWU)* of an itemset is the sum of the *transaction utility* of all transactions in which the itemset *X* is present. The transaction weighted utilization of an itemset *X* in the database *D* is denoted by *TWU*(*X*), and defined as,

$$\begin{array}{c} TWU\left(X\right)=\sum_{X\subseteq T_{c}\in D}TU\left(T_{c}\right) \end{array}$$ (6)

An itemset *X* in a database *D* is a high transaction weighted utility (*HTWUI*), if its *TWU* is greater than or equal to the user specified minimum threshold, where the minimum threshold *TU* is multiplied by threshold ratio *δ* as;

$$\begin{array}{c} HTWUI\leftarrow \{X|TWU(X)\geq TU\times \delta \} \end{array}$$ (7)

The *transaction utility* can be calculated for transactions *T_1_ = U(A, T_1_) + U(C, T_1_) + U(D, T_1_) = 8, T_2_ = U(A, T_2_) + U(C, T_2_) + U(E, T_2_) + U(G, T_2_) = 27*, similarly, *T_3_ = 55, T_4_ = 20, and T_5_ = 11* from the transaction database shown in Tables 1 and 2, respectively. The *transaction weighted utilization (TWU)* of itemset (A, C) can be calculated as follows, *TWU(A, C) = TU(T_1_) + TU(T_2_) + TU(T_3_) = 90*. The transaction weighted utilization of other itemsets can be calculated in a similar manner. The high utility mining uses another important property known as, an *anti-monotone property*, to prune the search space used in the Apriori algorithm [2]. For any itemset, if *TWU(X) < min.util*, then, *X* is a low utility itemset including all of its supersets. Many efficient algorithms are developed to find the high utility itemsets using the same property for pruning the search space. The algorithms such as Two Phase [9], UP-Growth [13], and UP-Growth+ operate in two phases. In the first phase, these algorithms find the candidate high utility itemsets, and filter out the low utility itemsets to find the exact high utility itemsets by scanning the transaction database multiple times. More efficient algorithms are developed recently, which calculates the high utility itemsets in a single phase. The algorithms like HUI-Miner [16], EFHM [21], and FHM [22] work in a single phase to find the exact high utility itemsets. We calculate the utility of the itemsets based on the *High Frequency*, and *Low Frequency* itemsets generated using the *FP-Growth* algorithm [3].

### Our contributions

Our aim is to design a framework, which generates different association rules for different combination of itemsets. The itemsets are generated based on the frequency as well as utility, hence, we can get more valuable association rules from these itemsets. We integrate the concept of *Frequency Itemset Mining*, and *Utility Itemset Mining* to generate the four type of itemsets, and eventually the association rules. We use the FP-Growth algorithm [3] to generate different type of itemsets, since the FP-Growth works in a single phase, and does not require multiple scan of transaction database. The FP-Growth algorithm [3] can be modified to generate different type of itemsets, and these itemsets can be used to generate different association rules. The key features of our contribution include the following major aspects:

1. Initially, we need to derive the 1 − *itemsets* from the transaction database and derive their frequency, and utility values.
2. After all the 1 − *itemsets* are derived from transaction database, we generate *k* − *itemsets* by using the *FP-tree* created for the FP-Growth algorithm [3]. We classify these itemsets as *High Frequency*, or *Low Frequency itemsets* based on the frequency value *min*_*supp* of *k* − *itemsets*.
3. Once the *High Frequency* and *Low Frequency k-itemsets* are generated by using the FP-Growth algorithm [3], we classify these itemsets into four different type of itemsets based on the utility value *min*_*util* of those itemsets. The four type of itemsets are as follows:
  - High Frequency High Utility HFHU itemsets
  - High Frequency Low Utility HFLU itemsets
  - Low Frequency High Utility LFHU itemsets
  - Low Frequency Low Utility LFLU itemsets
4. After the generation of four type of itemsets, we derive the association rules for the different combination of these itemsets based on the *Confidence* *min*_*conf* measure.

**Fig 1:**
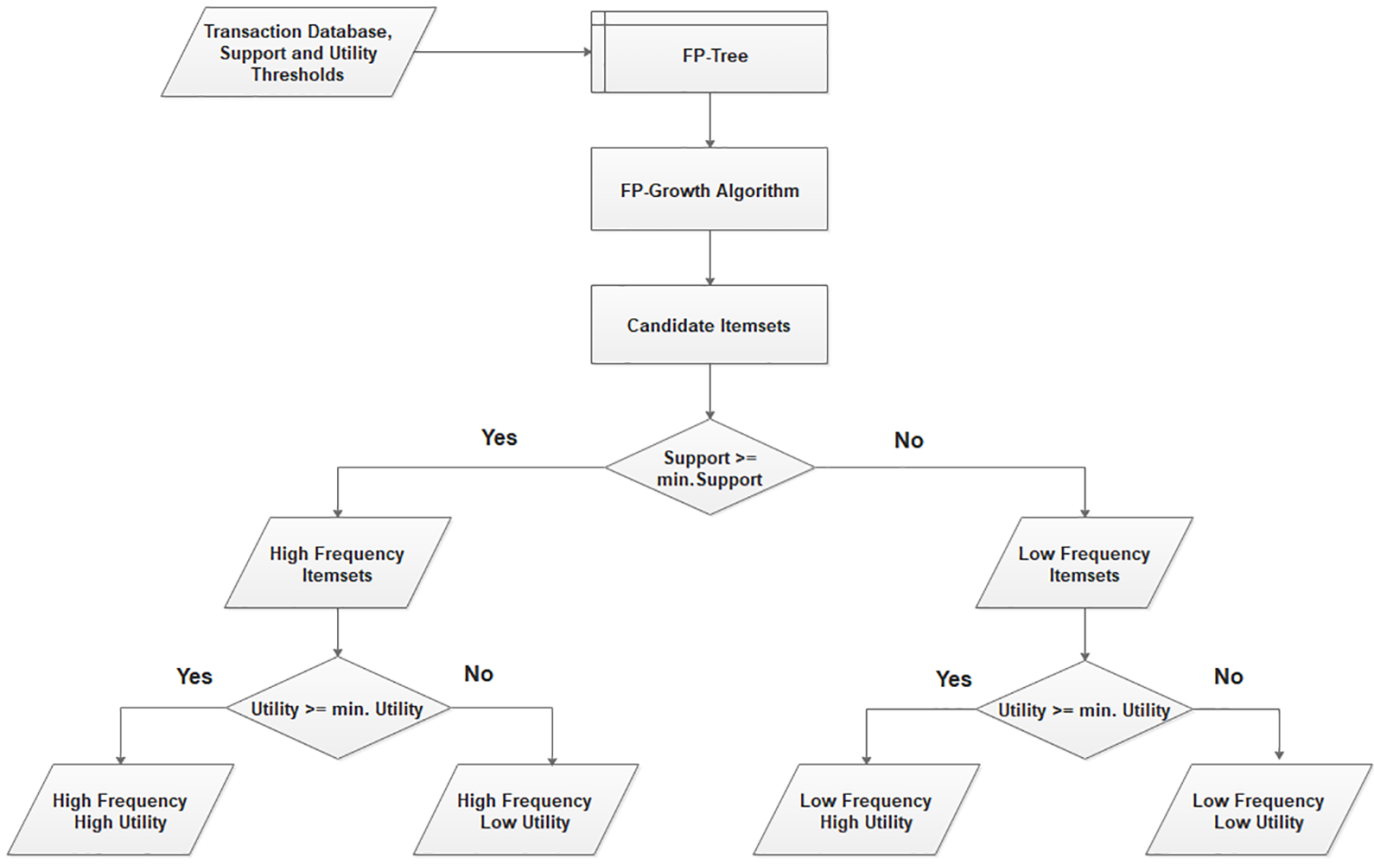
Phase 1—Proposed method.

**Fig 2:**
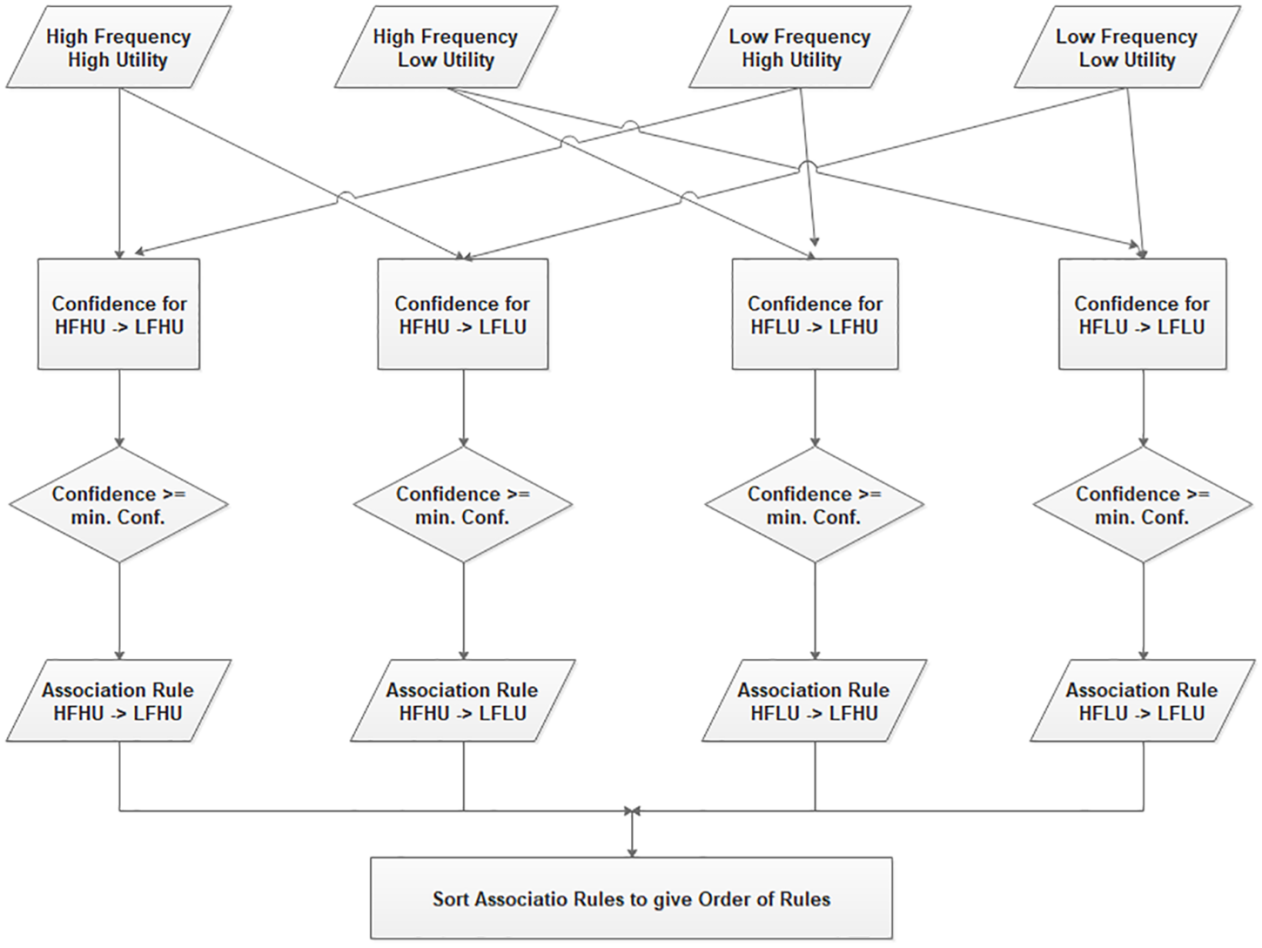
Phase 2—Proposed method.

Whole process is summarized in two phases, in the first phase four different type of itemsets *HFHU, HFLU, LFHU*, and *LFLU* can be generated by using modified FP-Growth algorithm [3]. Fig 1 represents the first phase of the process. In second phase, the association rules for the combination of high frequency itemsets with the low-frequency itemsets can be generated. Fig 2 represents the second phase of the process.

## Proposed combination approach

In this section, we define a method to combine the low-frequency itemsets with the high frequency itemsets [27–29], both having low, or high utility to generate new association rules.

### Problem statement

The frequency and utility are important measures in mining useful information from a transaction database. However, the high utility, or high frequency can not be the sole measure in mining this important information from a transaction database. The combination of frequency with utility can be useful to extract more valuable information. Our proposed algorithm combines the low-frequency itemsets with high-frequency itemsets, both having low, or high utility to derive different association rules. These association rules can be used by supermarkets, or retail stores to increase sales, and hence the profit from rarely sold items, which may or may not have high utility values.

#### Definitions

Consider the example of Tables 1 and 2, let *D = (T_1_, T_2_, T_3_,….. T_*m*_)* be a transaction database, and *I = (i_1_, i_2_, i_3_…i_*n*_)* be a set of all the items in the database. The transaction *T_*c*_ ∈ D* is a subset of *I* with a distinct identifier *TID*. For a transaction *T_*c*_*, each item is associated with a positive integer in the utility table known as *external utility*, and denoted as *p(i_*k*_, T_*c*_)*. Also, each item in a transaction *T_*c*_* is associated with a positive integer, known as *internal utility*, or *quantity utility*, and denoted as *q(i_*k*_, T_*c*_)*.

#### Definition 1

The *Frequency Itemset Mining* uses an important measure known as the *support* of an itemset *X*. The *support* of an itemset *X* is defined as, the *frequency* of an item or itemset in all transactions (number of times an item or itemset present in all transactions), divided by the total number of transactions *N(D)* in the database *D*, and is denoted as *supp(X)*.

$$\begin{array}{c} supp(X)=Supp.count(X)/N(D) \end{array}$$ (8)

#### Definition 2

The *Association Rule Mining (A,B) → C* uses an important measure known as the *confidence* of an itemset *(A,B,C) ∈ X*. The *Confidence* of an itemset *X* is defined as, the conditional probability of the *frequency* of a final itemset *(A, B, C)* derived from an underlying itemset *(A, B)*, with the *frequency* of an underlying itemset *(A, B)* in all transactions, and is denoted as *Conf(X)*.

$$\begin{array}{c} Conf(X)=Supp.count(A,B,C)/Supp.count(A,B) \end{array}$$ (9)

#### Definition 3

The *utility* of an itemset in a transaction is defined as, the *internal utility of an itemset × external utility* of an itemset, and is denoted as follows:

$$\begin{array}{c} u\left(X,T_{c}\right)=\sum_{i\in X\cap X\in T_{c}}p\left(i\right)\times q\left(i,T_{c}\right) \end{array}$$ (10)

#### Definition 4

An itemset is known as a *High Utility Itemset*, if it has the *utility* no less than a user specified *minimum utility threshold*, and is denoted as *min.Util*. Otherwise, an itemset is known as *low utility itemset*.

### Mining Association rules for Low Frequency itemsets

In this section, the proposed algorithm is described in detail to derive the association rule for the low-frequency itemsets in a transaction database. We use the FP-Growth algorithm [3] to generate the candidate itemsets, find the frequency, utility, and then generate different association rule for all the candidate itemsets. The FP-Growth algorithm [3] creates a novel tree structure known as *FP-tree* to generate the candidate itemsets. The FP-Growth algorithm [3] also calculates the support and confidence values. The association rules are generated based on the *min*.*Support* and *min*.*Confidence*, to define the association between different candidate itemsets. Our proposed method uses the same FP-Growth and *FP-tree* approach to generate different association rule for the combination of low-frequency, and high frequency itemsets. The utility of candidate itemsets should also be considered to generate these association rules. The utility of each itemset can be calculated while generating and calculating their support, and confidence values. The detailed explanation of how to generate different type of itemsets based on the combination of utility, and frequency is as follows:

#### Low frequency itemsets

The key contribution of this algorithm is to generate maximum possible rules to increase the frequency, utility, or both for the low-frequency as well as the high frequency itemsets. It is necessary to generate different combination of itemsets to find the desired association rules based on the frequency of itemsets. The FP-Growth algorithm [3] creates the *FP-tree* structure to generate the frequent itemsets, and remove the low-frequency itemsets. We create the same *FP-tree* structure without pruning the low-frequency itemsets, and use the same *FP-tree* to generate high frequency as well as low-frequency itemsets. An itemset is considered as a high frequency itemset (*HF*_*k*_), if its frequency is greater than or equal to *min*.*Support* which is denoted as *min*_*Sup* i.e minimum frequency threshold value.

$$\begin{array}{c} HF_{k}=C_{k}\subseteq T_{c}|Frequency\left(C_{k}\right)\geq min\_Sup \end{array}$$ (11)

An itemset is considered as a low-frequency itemset (*LF*_*k*_), if its frequency is less than *min*_*Sup* i.e minimum frequency threshold value.

$$\begin{array}{c} LF_{k}=C_{k}\subseteq T_{c}|Frequency\left(C_{k}\right)<min\_Sup \end{array}$$ (12)

#### FP-tree

Since we use the FP-Growth algorithm [3] to generate the candidate itemsets, it is necessary to discuss the *FP-tree* structure used for the generation of candidate itemsets. The *FP-tree* is a novel structure which stores items and their frequencies, and helps to create the conditional pattern base useful for the generation of candidate itemsets, without scanning the transaction database multiple time. Original FP-Growth algorithm [3] creates the *FP-tree* by pruning the low-frequency itemsets. However, for our purpose, we do not prune the low-frequency itemsets and include them in the *FP-tree* structure. Thus, the conditional pattern base for every item contains low-frequency as well as high frequency itemsets. Since we store the support value of every item in *FP-tree* structure, this support information can be used to classify the itemsets into *High Frequency*, or *Low Frequency* itemsets.

#### FP-growth

Construction of a compact *FP-tree* ensures that subsequent mining can be performed with a rather compact data structure. The FP-Growth algorithm [3] is used to generate the candidate itemsets by exploring the compact information stored in the *FP-tree*. The FP-Growth mining process scans the *FP-tree* once and generate a conditional pattern base for each item *C*_*i*_ in the transaction database. The conditional pattern base of each item contains a set of transformed prefix paths having all the items, which share the same transaction number, and support value as *C*_*i*_. The itemset mining is then recursively performed on the conditional pattern base of each item *C*_*i*_ by constructing a *conditional FP-tree*. This *conditional FP-tree* is usually much smaller than original tree, and is bounded by maximum depth of the *FP-tree*. Moreover, the itemset mining operation consists of prefix count adjustment, counting the frequency of item, and concatenation of items to form low-frequency, or high frequency itemset. This is much less costly compared to candidate itemset generation in Apriori algorithm, thus, the proposed algorithm is efficient.

#### Calculate utility

It is necessary to calculate the utility of each candidate itemset generated as above (*HF*_*k*_ and *LF*_*k*_) based on the utility value assigned to every item in the utility table in Table 2. Once the low-frequency and high frequency itemsets are generated using the *FP-tree* structure, the utility of each itemset can also be calculated based on the following formula.

$$\begin{array}{c} Utility\left(C_{k}\right)=\sum_{i_{j}\subseteq C_{k}\cap C_{k}\subseteq T_{c}}Frequency\left(i_{j}\right)\times Utility\left(i_{j}\right) \end{array}$$ (13)

It is necessary to consider the utility value of each item in each transaction of utility table, to calculate the utility of each itemset. Thus, we create an index structure *I*, where, utility of every item *Utility*(*C*_*k*_) is stored with the corresponding transaction number *T*_*c*_. For each item *X* ⊂ *C*_*k*_, the corresponding list *Trans*(*X*) of all transactions is derived and the common transactions are derived using *AND* operation on those lists. Thus, the utility value *Utility*(*C*_*k*_) is derived by adding the utility value of every item in the common transaction. The index structure *I* help to reduce the multiple scan of the utility table, and we can easily get the utility value of itemsets from the index structure. If the *utility* *of* *C*_*k*_ is greater than or equal to *min*.*Utility*, then the candidate itemset *C*_*k*_ is a high utility itemset, otherwise, it is a low utility itemset. Based on the definition of utility of an itemset, we generate different combination of the low utility and high utility itemsets with the low-frequency and high frequency itemsets as follows: If the utility of a high frequency itemset (*HF*_*k*_) is greater than or equal to *min*_*util*, then it is considered as the *High Frequency High Utility* itemset (*HF*_*k*_*HU*_*k*_).

$$\begin{array}{c} HF_{k}HU_{k}=C_{k}\in HF_{k}|u\left(C_{k}\right)\geq min\_util\; \end{array}$$ (14)

If the utility of a high frequency itemset (*HF*_*k*_) is less than *min*_*util*, then it is considered as the *High Frequency Low Utility* itemset (*HF*_*k*_*LU*_*k*_).

$$\begin{array}{c} HF_{k}LU_{k}=C_{k}\in HF_{k}|u\left(C_{k}\right)<min\_util\; \end{array}$$ (15)

If the utility of a low-frequency itemset (*LF*_*k*_) is greater than or equal to *min*_*util*, then it is considered as the *Low Frequency High Utility* itemset (*LF*_*k*_*HU*_*k*_).

$$\begin{array}{c} LF_{k}HU_{k}=C_{k}\in LF_{k}|u\left(C_{k}\right)\geq min\_util\; \end{array}$$ (16)

If the utility of a low-frequency itemset (*LF*_*k*_) is less than *min*_*util*, then it is considered as the *Low Frequency Low Utility* itemset (*LF*_*k*_*LU*_*k*_).

$$\begin{array}{c} LF_{k}LU_{k}=C_{k}\in LF_{k}|u\left(C_{k}\right)<min\_util\; \end{array}$$ (17)

#### Pre-large threshold

Since we use the *FP-tree* structure to generate low-frequency as well as high frequency itemsets, and there is not any pruning criteria to reduce the number of itemsets which generate the least utility in the process, we need to define some criteria to eliminate itemsets which have the least confidence value of the association rules for different combination of itemsets. The concept of pre-large itemsets [30, 31], which defines a low support threshold, are used to prune itemsets having the least support values. Two pre-large thresholds are defined, one with the frequency, and another with the utility. The low support threshold for pre-large itemsets helps to prune the itemsets which have the support value less than the low support threshold, and hence requires less time to generate the candidate itemsets. Similarly, the low utility threshold for pre-large itemsets helps to prune the itemsets which have the utility less than the low utility threshold, and hence requires less time to generate the candidate itemsets.

#### Proposed algorithm

In this section, the proposed method is described based on the above definitions. Whole pseudo-code is divided into two algorithms, the Algorithm 1 generates the low-frequency and high frequency itemsets based on the FP-Growth method [3], and calculate the utility of these itemsets to generate four different type of itemsets. The Algorithm 1 is a modified version of the FP-Growth [3] algorithm, where the low-frequency itemsets are also considered in the construction of *FP-tree* structure. The *FP-tree* structure is used to generate a conditional base pattern for every item, which further produces all the candidates for high frequency as well as low-frequency itemsets. Once the candidate itemsets are generated, the utility values are calculated for all the itemsets to classify them in four type of itemsets. The Algorithm 2 generates different association rules for these four different type of itemsets generated by the Algorithm 1. The Algorithm 3 provides a basic method to generate different association rules for different type of itemsets.

**Algorithm 1** mining low frequency itemsets

**Input:**

*D*: transaction database;

*min*_*util*: minimum utility threshold;

*min*_*sup*: minimum frequency threshold;

*supp*: Support value of an item;

*conf*: confidence value of an association rule;

**Output:**

*HFHU*: High Frequency High Utility Itemset;

*HFLU*: High Frequency Low Utility Itemset;

*LFHU*: Low Frequency High Utility Itemset;

*LFLU*: Low Frequency Low Utility itemset;

1: **for** each Transaction *T*_*c*_ ∈ *DB* **do**

2:  **for** each item *C*_*i*_ ∈ *T*_*c*_ **do**

3:   *supp*(*C*_*i*_) = *count*(*C*_*i*_) + +;

4:  **end for**

5: **end for**

6: Sort *T*_*c*_ ∈ *DB* with *supp*(*C*_*i*_) in descending order

7: *insert*_*FP*_*tree*([*C*_*i*_|*T*_*c*_)

8: **for** each item *C*_*i*_ ∈ *DB* **do**

9:  generate candidate itemsets *C*_*k*_ = *FP*_*Growth*(*FP*_*Tree*, *C*_*i*_)

10: **end for**

11: *HF*_*k*_ = {*c* ∈ *C*_*k*_|*supp*(*C*_*k*_)≥ *min*_*sup*}: frequent itemset in *DB* in *k* scan;

12: *LF*_*k*_ = {*c* ∈ *C*_*k*_|*supp*(*C*_*k*_)< *min*_*sup*}: low-frequency itemset in *DB* in *k* scan;

13: **for** each item *C*_*k*_ ∈ *T*_*c*_ **do**

14:  *Utility*(*C*_*k*_) = ∑*Frequency*(*C*_*k*_ ∈ *T*_*c*_) × *Utility*(*C*_*k*_ ∈ *T*_*c*_);

15:  *HF*_*k*_*HU*_*k*_ = {*C*_*k*_ ∈ *HF*_*k*_|*Utility*(*C*_*k*_) ≥ *min*_*util*};

16:  *LF*_*k*_*LU*_*k*_ = {*C*_*k*_ ∈ *LF*_*k*_|*Utility*(*C*_*k*_) < *min*_*util*};

17:  *LF*_*k*_*LU*_*k*_ = {*C*_*k*_ ∈ *LF*_*k*_|*Utility*(*C*_*k*_) ≥ *min*_*util*};

18:  *HF*_*k*_*LU*_*k*_ = {*C*_*k*_ ∈ *HF*_*k*_|*Utility*(*C*_*k*_) < *min*_*util*};

19: **end for**

20: *Rules k* = *Algorithm 2* to generate Association rules for 4 types of itemsets;

21: **return** *Rule 1, Rule 2, Rule 3, Rule 4*

**Algorithm 2** association rules for low frequency itemsets

**Input:**

D: transaction database;

*HFLU*: High Frequency Low Utility itemset;

*HFHU*: High Frequency High Utility itemset;

*LFLU*: Low Frequency Low Utility itemset;

*LFHU*: Low Frequency High Utility itemset;

*Sup*: the minimum support threshold value;

*Conf*: the minimum confidence threshold;

**Output:** Association Rules:

*Rule* 1: *LFLU* → *HFHU*;

*Rule* 2: *LFHU* → *HFHU*;

*Rule* 3: *LFHU* → *HFLU*;

*Rule* 4: *LFLU* → *HFLU*;

1: **for** *C*_*k*_ ∈ *DB* **do**

2:  generate association rules for 4 types of itemsets with less frequent itemset as below;

3:  *Confidence* *of* *C*_*k*_ = *Support* *of* *C*_*k*−1_ ∈ *DB* ÷ *Support* *of* *C*_*k*_ ∈ *DB*

4:  *R*_1_ = *Sup*(*HF*_*k*_*HU*_*k*_) ÷ *Sup*(*LF*_*k*_*HU*_*k*_)

5:  Rule 1 = {*C*_*k*_ ∈ *HF*_*k*_*HU*_*k*_ → *LF*_*k*_*HU*_*k*_ | *if* *R*_1_ ≥ *min*_*conf*}

6:  *R*_2_ = *Sup*(*HF*_*k*_*LU*_*k*_) ÷ *Sup*(*LF*_*k*_*HU*_*k*_)

7:  Rule 2 = {*C*_*k*_ ∈ *HF*_*k*_*LU*_*k*_ → *LF*_*k*_*HU*_*k*_| *if* *R*_2_ ≥ *min*_*conf*}

8:  *R*_3_ = *Sup*(*HF*_*k*_*HU*_*k*_) ÷ *Sup*(*LF*_*k*_*LU*_*k*_)

9:  Rule 3 = {*C*_*k*_ ∈ *HF*_*k*_*HU*_*k*_ → *LF*_*k*_*LU*_*k*_| *if* *R*_3_ ≥ *min*_*conf*}:

10:  *R*_4_ = *Sup*(*HF*_*k*_*LU*_*k*_) ÷ *Sup*(*LF*_*k*_*LU*_*k*_)

11:  Rule 4 = {*C*_*k*_ ∈ *HF*_*k*_*LU*_*k*_ → *LF*_*k*_*LU*_*k*_| *if* *R*_4_ ≥ *min*_*conf*}

12: **end for**

13: **return** *Rule*1, *Rule*2, *Rule*3, *Rule*4

**Algorithm 3** association rules

**Input**: *Itemset*1, *Itemset*2, *min*.*Confidence*;

**Output**: *AssociationRule*: *Itemset*1 → *Itemset*2;

1: *X* ← *Itemset*1

2: *Y* ← *Itemset*2

3: **if** *X* ⊆ *Y* **then**

4:  *Confidence* ← *Support*(*X*) ÷ *Support*(*Y*)

5:  **if** *Confidence* ≥ *min*.*Confidence* **then**

6:   *Rule* ← *Itemset*2 → *Itemset*1

7:  **end if**

8: **end if**

9: **return** *Rule*

### Example of combination rules

Following example explains how the proposed algorithm generates four different kind of itemsets from a sample transaction database. The itemsets and the corresponding utility value of each item in the transaction database is showin in Table 3 as follows:

**Table 3:** Transaction database with profit values.

TID	Transaction	Utility of items
*T*_1_	A, B, C	3, 2, 2
*T*_2_	A, C, D	3, 3, 10
*T*_3_	A, B, C, F	5, 3, 4, 20

**Fig 3:**
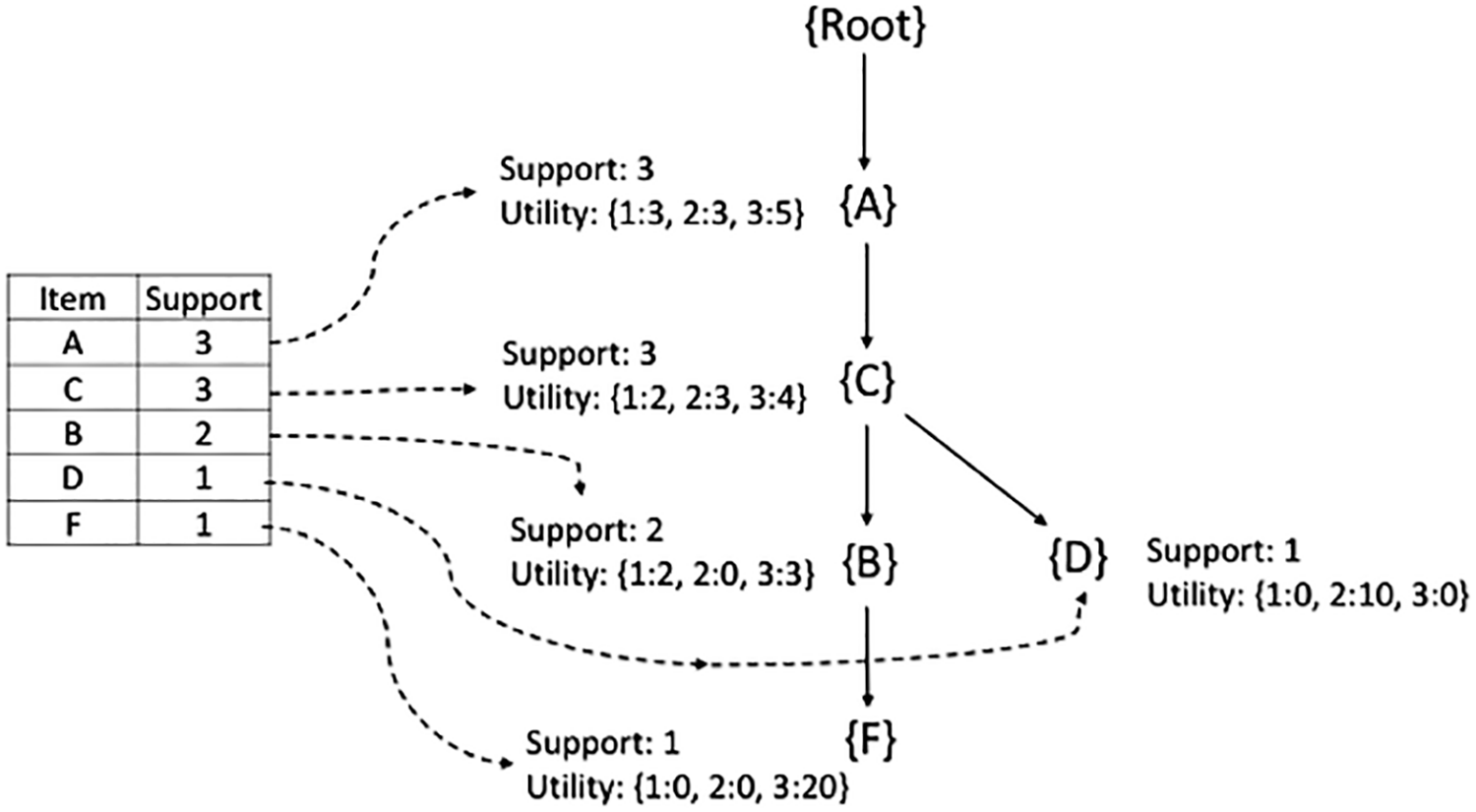
FP-tree for sample transaction database.

Fig 3 shows the *FP-tree* generated for the sample transaction database. Table 4 shows the candidate set of frequent itemsets with the support value ≥ *min.Support = 0.40* in a transaction database. Table 5 shows the candidate set of low-frequency itemsets with the support value less than *min.Support = 0.40*. After these two candidate itemsets are generated, we calculate the utility of these itemsets to categorize them as *High Utility*, or *Low Utility* as follows:

**Table 4:** Frequent itemsets (min. support ≥ 0.40).

Itemset	Support
(*A*)	1.0
(*B*)	0.66
(*C*)	1.0
(*A*, *B*)	0.66
(*A*, *C*)	1.0
(*C*, *B*)	0.66
(*A*, *C*, *B*)	1.0

**Table 5:** Less frequent itemsets (min. support < 0.40).

Itemset	Support
(*D*), (*F*)	0.33
((*A*, *D*)(*C*, *D*), (*A*, *F*), (*B*, *F*), (*C*, *F*))	0.33
((*A*, *B*, *F*), (*A*, *C*, *D*), (*A*, *C*, *F*), (*C*, *B*, *F*))	0.33
(*A*, *C*, *B*, *F*)	0.33

Table 6 shows *High Frequency High Utility* (*HFHU*) itemsets, i.e. high frequency itemsets having utility ≥ *min. Utility = 20*. Table 7 shows *High Frequency Low Utility* (*HFLU*) itemsets, i.e. high frequency itemsets having utility < *min. Utility = 20*. Table 8 gives *Low Frequency High Utility* (*LFHU*) itemsets, i.e. low-frequency itemsets having utility ≥ *min. Utility = 20*. Table 9 gives *Low Frequency Low Utility* (*LFLU*) itemsets, i.e. low-frequency itemsets having utility < *min. Utility = 20*.

**Table 6:** High frequency high utility (*HFHU*) itemsets.

Itemset	Utility
(*A*, *C*)	20

**Table 7:** High frequency low utility (*HFLU*) itemsets.

Itemset	Utility
(*A*)	11
(*B*)	5
(*C*)	9
(*A*, *B*)	13
(*C*, *B*)	11
(*A*, *C*, *B*)	19

**Table 8:** Low frequency high utility (*LFHU*) itemsets.

Itemset	Utility
(*F*)	20
(*A*, *F*)	25
(*B*, *F*)	23
(*C*, *F*)	24
(*A*, *B*, *F*)	28
(*A*, *C*, *F*)	29
(*C*, *B*, *F*)	27
(*A*, *C*, *B*, *F*)	32

**Table 9:** Low Frequency Low Utility (*LFLU*) itemsets.

Itemset	Utility
(*D*)	10
(*A*, *D*)	13
(*C*, *D*)	13
(*A*, *C*, *D*)	16

### Association rules order

**Fig 4:**
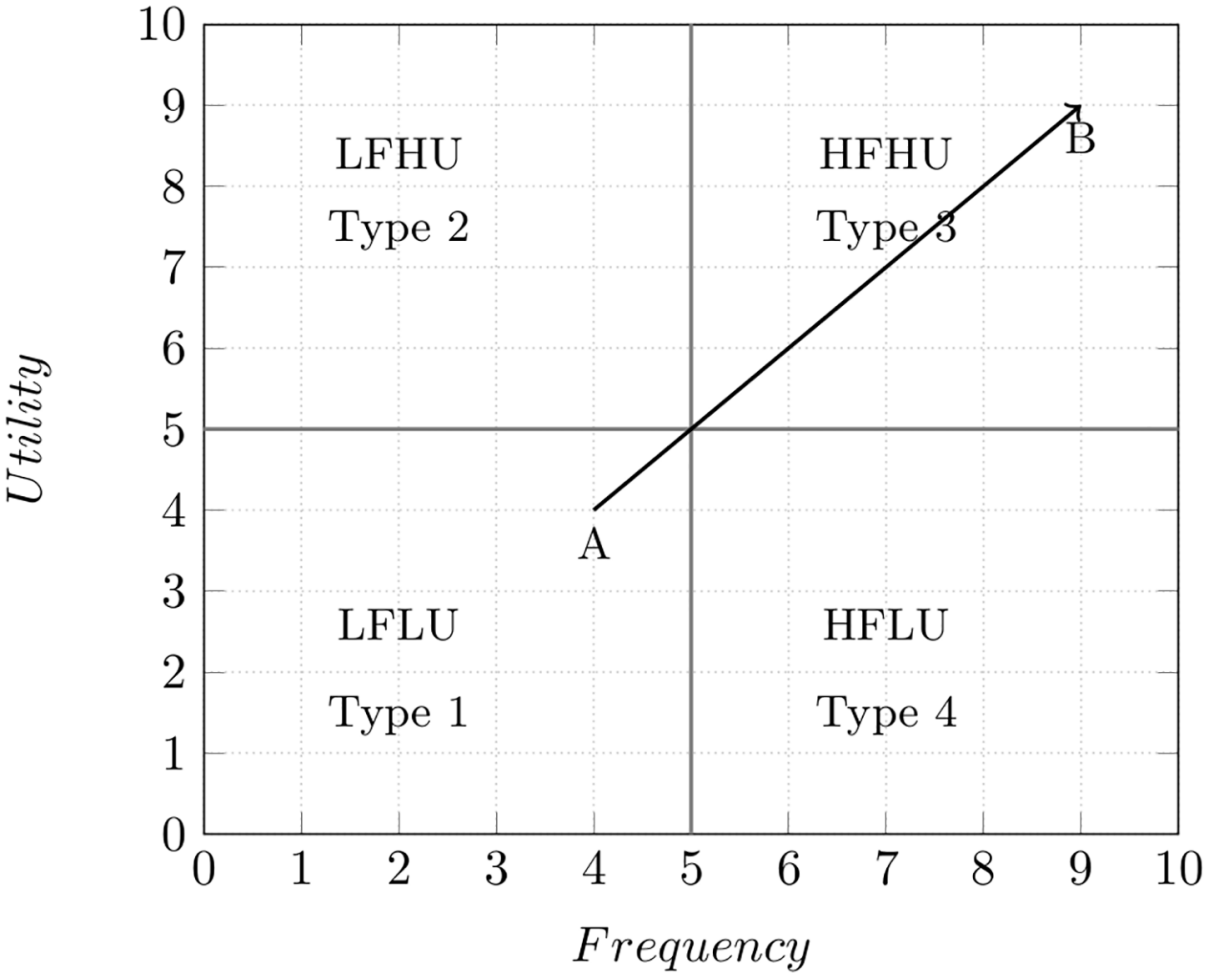
Association rules order.

Four different type of association rules can be derived for low-frequency itemsets from the algorithm described in the previous sections. First, we find the (*Low Frequency (LF)* itemsets) along with their utility. If they have utility value greater than or equal to *min_util* value, then they can be classified as *Low Frequency High Utility (LFHU)* itemsets. If they have utility value less than *min_util* value, then they can be classified as *Low Frequency Low Utility (LFLU)* itemsets. These four type of itemsets can be plotted on a graph to depict the different set of combinations considered while deriving these association rules, as follows. The X- axis represents the *frequency* of itemsets, and Y- axis represents the *utility* of itemsets. Based on the Fig 4, the first quadrant of the graph represents the *Type 1* *Low Frequency Low Utility (LFLU)* itemsets. The second quadrant of the graph represents the *Type 2* *Low Frequency High Utility (LFHU)* itemsets. The third quadrant of the graph represents the *Type 3 High Frequency High Utility (HFHU)* itemsets. And finally, the fourth quadrant of the graph represents the *Type 4 Low Frequency Low Utility (LFLU)* itemsets. The association rules generated for all 4 different itemsets are as follows:

#### Low Frequency Low Utility → High Frequency High Utility

Table 10 shows the association rules generated for *LFLU* → *HFHU* from example database presented in Tables 1 and 2, respectively. These type of association rules can generate the maximum utility for the low utility itemsets, or can increase the frequency of the low-frequency itemsets. If we combine the *low-frequency* itemset having low utility *(LFLU)*, with the frequently sold itemset with high utility *(HFHU)*, we can increase the utility of *LFLU* itemset, and frequency of *LFLU* itemset. Following list of suggestions can be provided for the above list of association rules:

**Table 10:** Association rules for *LFLU* → *HFHU*.

Association Rule	Confidence
(*A*, *C*) → (*A*, *C*, *D*)	33.33

Suggestions:

- The low-frequency *(LFLU)* itemsets can be grouped together with the high frequency *(HFHU)* itemsets at the same place in super market, or retail stores to increase frequency of the low-frequency *(LFLU)* itemsets.
- Discount offers, like *Buy One, Get One Free*, can be provided on the low utility itemsets, so that sale of the combination of *LFLU* and *HFHU* itemsets can be increased.
- Discount offers, like *20-30% off*, can be provided on the high utility *(HFHU)* itemsets, so that the frequency of *LFLU*, and utility of *LFLU*, or *HFHU* itemsets can be increased.

#### Low Frequency High Utility → High Frequency High Utility

Table 11 shows the association rules for *LFHU* → *HFHU* generated from example database represented in Tables 1 and 2, respectively. With these type of association rules, we can get the combination of the *Low Frequency High Utility (LFHU)* itemset with the *High Frequency High utility (HFHU)* itemset. If the low-frequency itemsets having high utility *(LFHU)*, are combined with the frequently sold itemsets having high utility *HFHU)*, the frequency of *LFHU* itemset, and the utility of *LFHU*, and *HFHU* itemsets can be increased. Following list of suggestions can be provided for the above association rules:

**Table 11:** Association rules for *LFHU* → *HFHU*.

Association Rule	Confidence
(*A*, *C*) → (*A*, *C*, *F*)	33.33
(*A*, *C*) → (*A*, *C*, *B*, *F*)	33.33

Suggestions:

- The low-frequency *LFHU* itemsets can be grouped together with the high frequency *HFHU* itemsets at the same place in super market, or retail stores to increase frequency of *LFHU* itemsets.
- Discount offers, like *20-30% off*, can be provided on the high utility *(LFHU, HFHU)* itemsets when the combination of *(LFHU)* and *(HFHU)* itemsets is purchased. In this way, it can increase the frequency of *(LFHU)* itemsets, and the utility of *LFHU* and *HFHU* itemsets.

#### Low Frequency High Utility → High Frequency Low Utility

Third type of association rule is the combination of the *Low Frequency High Utility (LFHU)* itemset with the *High Frequency Low utility (HFLU)* itemset, generated from example database represented in Tables 1 and 2, respectively. Table 12 represents the association rule generated for the combination of *LFHU* → *HFLU* itemsets. If we combine the low-frequency itemset having high utility *(LFHU)*, with the frequently sold itemset having low utility *(HFLU)*, following list of suggestions can be provided to increase the frequency of low-frequency itemsets, and utility of the low utility itemsets.

**Table 12:** Association rules for *LFHU* → *HFLU*.

Association Rule	Confidence
(*A*, *B*) → (*A*, *B*, *F*)	33.33
(*A*, *B*) → (*A*, *C*, *B*, *F*)	33.33
(*A*, *C*) → (*A*, *C*, *F*)	25.0
(*A*, *C*) → (*A*, *C*, *B*, *F*)	25.0
(*A*, *C*, *B*) → (*A*, *C*, *B*, *F*)	33.33

Suggestions:

- The low-frequency itemsets *(LFHU)* can be grouped together with the high frequency itemsets *(HFLU)* at the same place in super market, or retail stores. In this way, we can increase the frequency of low-frequency itemsets.
- Discount offers, like *Buy One, Get One Free*, can be provided on the low utility itemsets *(HFLU)*, so that the frequency of the combination of *LFHU* and *HFLU* itemsets can be increased. This will help to increase utility of the low utility *(HFLU)*, and frequency of the low-frequency *(LFHU)* itemsets.
- Discount offers, like *20-30% off*, can be provided on the high utility *(LFHU)* itemsets on the purchase of the combination of *HFHU*, and *LFLU* itemsets. In this way, the frequency of *(LFHU)* itemsets, and utility of *HFLU* itemsets can be increased.

#### Low Frequency Low Utility → High Frequency Low Utility

Fourth type of association rule is the combination of the *Low Frequency Low Utility (LFLU)* itemsets with the *High Frequency Low utility (HFLU)* itemsets, generated from example database represented in Tables 1 and 2, respectively. Table 13 shows the association rule for the combination *LFLU* → *HFLU* itemsets. If the low-frequency itemsets having low utility *(LFLU)* are combined with the frequently sold itemset having low utility *(HFLU)*, the frequency of the low-frequency itemsets can be increased. Following list of suggestions can be provided to generate the high frequency, and high utility for the combination of itemsets.

**Table 13:** Association rules for *LFLU* → *HFLU*.

Association Rule	Confidence
(*A*) → (*A*, *D*)	33.33
(*A*) → (*A*, *C*, *D*)	33.33
(*C*) → (*C*, *D*)	33.33
(*C*) → (*A*, *C*, *D*)	33.33

Suggestions:

- The low-frequency *LFLU* itemsets can be grouped with the high frequency *HFLU* itemsets at the same place in the super market, or retail stores. This will help to increase the frequency of low-frequency itemsets.
- Discount offers, like *Buy One, Get One Free*, can be provided on the itemsets having the lowest utility among all itemsets, so that the frequency of combination of *LFLU* and *HFLU* itemsets can be increased. This will help to increase the frequency of low-frequency itemsets.

When the process is executed using the sample transaction databases shown in Tables 1 and 3, respectively, a different set of association rules are generated. Tables 14 and 15 shows the number of association rules generated from sample *Databases 1 and 2*.

**Table 14:** Number of rules for sample database 1.

Database 1	Number of Rules
*HFHU* → *LFHU*	15
*HFLU* → *LFHU*	68
*HFHU* → *LFLU*	3
*HFLU* → *LFLU*	4

**Table 15:** Number of rules for sample database 2.

Database 2	Number of Rules
*LFLU* → *HFHU*	1
*LFHU* → *HFHU*	2
*LFHU* → *HFLU*	17
*LFLU* → *HFLU*	4

#### Order of rules

From Fig 4, it can be inferred that, there may be few itemsets which can be easily transformed from *LFLU* *A* to *HFHU* *B* by simply adding *HFHU* *itemset* to *LFLU* *itemset*. Thus, it is necessary to define the order of the association rules, which can be more useful to define different business strategies based on the requirements. If an association rule can increase the frequency as well as the utility of the low-frequency itemset, then that association rule will have more priority. Otherwise, the association rule which can only increase the frequency of low-frequency itemset will have less priority compared to the earlier rule. The total ordering, denoted by ≻, is the ordering of the association rules in terms of utility value. The rules with the higher utility have the highest priority, compared to the rest of the rules. We can denote the order of 4 type of association rules as follows:

$$\begin{array}{c} LFLU\to HFHU≻LFHU\to HFHU≻LFHU\to HFLU≻LFLU\to HFLU \end{array}$$

Based on these association rules, different businesses can decide different strategies like discount offers, or group the less frequently sold items with frequently sold items to increase the sale and eventually profit of the less frequently sold items.

## Algorithm analysis

Since the proposed approach is implemented using the FP-Growth algorithm [3] to derive different combination of itemsets, there are various factors impacting the computational complexity of the proposed method. The proposed method considers the low-frequency as well as high frequency itemsets, hence, there is no major pruning criteria required in this method. As per the proposed method, it is necessary to consider all the combination of candidate itemsets in the same transaction to generate four type of itemsets, and then generate association rule for those itemsets. The computational complexity analysis of the proposed method is described in detail as follows:

### FP-tree creation

The first step in the proposed method is to derive the 1-itemsets from the transaction database, and create the *FP-tree*. This step requires a single scan of transaction database. If we assume that, there are *m* number of transactions and average *n* items per transaction in the database, then 1-itemsets and *FP-tree* creation require *O*(*mn*) time. Since we do not prune the low-frequency items, we consider all the items while creating *FP-tree* from a transaction database. Hence, the time required is based on the number of items per transaction, which is *O*(*mn*).

### Generation of candidate itemsets

Once the *FP-tree* is created for all items from a transaction database, the next step is to derive conditional pattern base for all items, and then generate low-frequency as well as high frequency itemsets. The conditional pattern base is created for every item based on the path from the root of *FP-tree*. The conditional pattern base also takes into account the maximum support of an item, and include all the items in the prefix-path of a given item having a similar support value. Thus, the candidate itemset generation requires repeated scanning of the conditional pattern base, and requires the time as follows:

$$\begin{array}{c} C_{k+1}=\sum_{i=1}^{n}supp\left(C_{i}\right)+\sum_{A=frequent}\sum_{j=1}^{n}supp\left(A\cup C_{j}\right)+\sum_{B=low-frequency}\sum_{k=1}^{n}supp\left(B\cup C_{k}\right) \end{array}$$ (18)

Since we consider the low-frequency as well as high frequency itemsets, and the conditional pattern base for every item is scanned multiple times to generate both type of itemsets, the computational complexity will involve the every path of every item in *FP-tree*. The maximum depth of any path is bounded by *m* for *FP-tree*, and there can be *m* maximum scans for all items. Thus, the time required to generate all candidate itemsets is bounded by complexity *O*(*m*^2^).

### Calculate utility of itemsets

After the candidate itemset generation, next step is to calculate the utility value for each itemset, and classify those itemsets as the low-utility, or high utility itemsets. The utility value for each item in each transaction is stored in an index structure *I*. This index structure contains the information regarding the utility value of every item *Utility*(*C*_*k*_), and the corresponding transaction number *T*_*c*_(*C*_*k*_). For every item in an itemset, we retrieve the utility index for that item from the index structure, and find the common transactions and utility value of two or more items in an itemset. This comparison requires some constant amount *w* for all itemsets and involves *n* items, and almost *m* utility value for each item in an index structure. Hence, the total complexity for calculating the utility of candidate itemsets and classifying them in four type of itemsets can be given as, *O*(*mnw*), which is *O*(*mn*).

### Generation of association rules

Next phase is to derive the association rules for the different combination of itemsets. The association rules are derived by comparing the itemsets from four different categories based on the common factor in the two itemsets, and the *Confidence* measure *min*_*conf*. Suppose there are *K* itemsets in each type of itemset, then $\sum_{i=1}^{K}i\sum_{j=1}^{K}j$ comparisons are required to generate all the association rules. Thus, the total complexity for the proposed method can be expressed as below:

$$\begin{array}{c} Time\left(AssociationRules\right)=\sum_{i=1}^{n}\sum_{k=1}^{m}C_{k}\sum_{i=1}^{m}C_{i}\sum_{j=1}^{m}C_{j}=m\left(n^{2}\right)K^{2} \end{array}$$ (19)

$$\begin{array}{c} Totaltime=O\left(mn\right)+O\left(m^{2}\right)+O\left(mn\right)+O\left(mn^{2}k^{2}\right)=O\left(mn^{2}w\right)=O\left(mn^{2}\right) \end{array}$$ (20)

## Experimental results

We perform different experiments to find the association rules for the different combination of itemsets generated using the proposed method (Mining Association rules for Low Frequency itemsets). These experiments are executed on Intel Core i5 @ 3.70GHz, and Windows 7 operating system with 64 GB main memory. The algorithms are implemented in Python 2.7.

### Incremental experiments on small datasets

**Fig 5:**
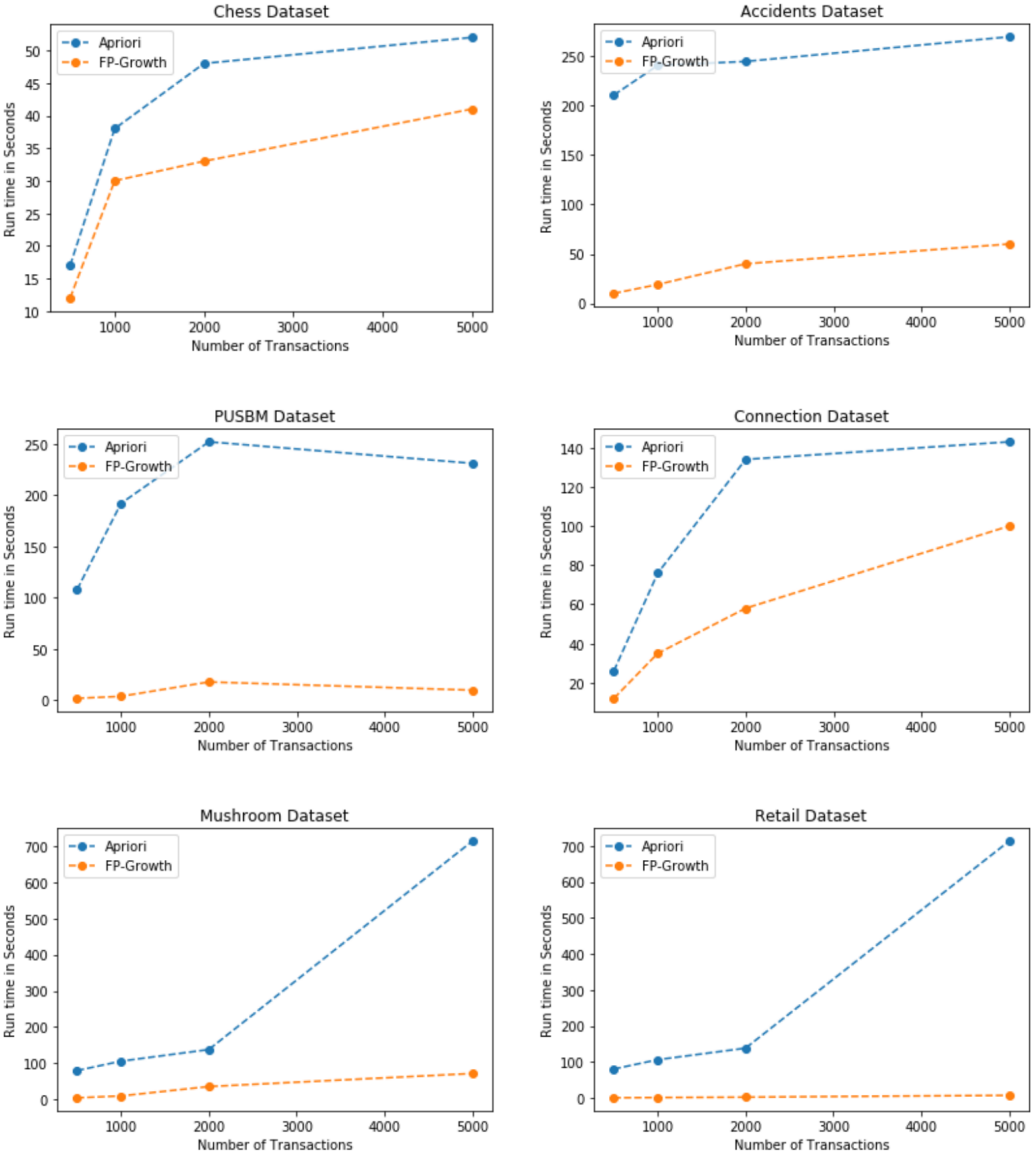
Experimental results on small datasets.

The real world datasets [32] are used for the experiment to generate different association rules. Four different type of itemsets are generated by the proposed algorithm based on the input datasets. The real world datasets (Chess, Connect, PUMSB, Accidents, Mushroom, and Retail) proves the authenticity of the proposed algorithm on the real world data. Initially, we perform the experiment on the small scale datasets. Since we use the FP-Growth algorithm [3] to generate the low-frequency as well as high frequency itemsets, there is not any pruning criteria involved in the whole process. Thus, the low support and low utility threshold value of pre-large itemsets [30, 31] can be used to prune certain itemsets, which have the least utility, or frequency for different combination of itemsets. We also perform these experiments by partitioning the transaction database into incremental value of the number of transactions, and 10 items per transaction. We iteratively perform the experiment on the datasets having *N = 500, 1000, 2000 and 5000* transactions with 10 items per transaction. Since the Apriori algorithm [2] works in multiple phases, and the FP-Growth [3] works in single scan of transaction database, we need to compare both the implementations to prove the authenticity of the proposed method. The experimental results shown in Fig 5 compare the two experiments, and proves that the FP-Growth [3] implemenation of the proposed approach is more efficient than Apriori implementation, and provide more valuable association rules for low-frequency as well high frequency itemsets.

### Incremental experiments on large datasets

**Fig 6:**
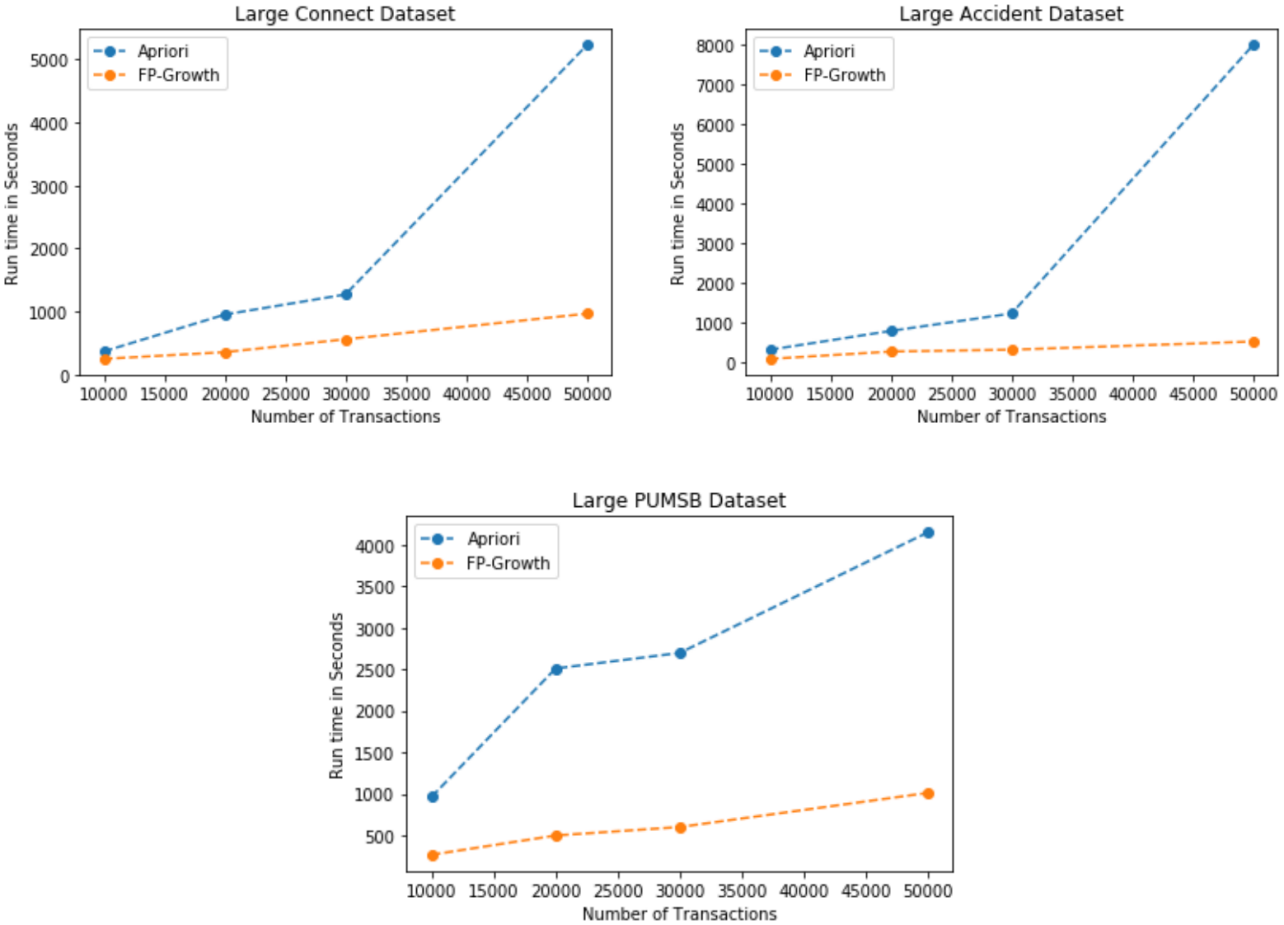
Experimental results on large datasets.

We use the same real world datasets [32] to verify the efficiency of the proposed method using the FP-Growth [3], and Apriori algorithm [2] on the large datasets. The large datasets (Connect, PUMSB, Accidents, and Retail) are used for the experiment. We perform all the experiments iteratively using the datasets having *N = 10,000, 20,000, 30,000 and 50,000* transactions, and 10 itemsets per transaction. The experimental results for the large datasets are shown in Fig 6. Different values of *min*_*sup*, *min*_*util* and *min*_*conf* are used for all the experiments on small as well as large datasets, and the number of association rules are also recorded for each experiment on each transaction dataset. The overall statistics of all the datasets used in small as well as large-scale experiment are shown in Table 16. The experimental results for the iterative experiments show different association rules generated for different combination of itemsets. The association rules for the low-frequency itemsets from a real world data shows that different type of relations, or information can be extracted from the large volume of data. These association rules can help different businesses to provide discount offers to increase sale, and eventually profit from the low-frequency items.

**Table 16:** Number of rules for all experiments.

Dataset	Transactions	Association Rules	
D	N	Apriori (n = 10)	Fp-Growth (n = 10)
Accidents	500	2022	1921
Accidents	1000	2012	1890
Accidents	2000	505	1250
Accidents	5000	686	1425
Accidents	10000	4752	4400
Accidents	20000	5950	6260
Accidents	30000	5020	4871
Accidents	50000	362	4389
Connect	500	2080	2043
Connect	1000	811	815
Connect	2000	3240	840
Connect	5000	1600	5971
Connect	10000	10505	9430
Connect	20000	9020	4233
Connect	30000	5200	4580
Connect	50000	4470	4805
PUMSB	500	511	475
PUMSB	1000	42	70
PUMSB	2000	48	80
PUMSB	5000	455	411
PUMSB	10000	15870	15903
PUMSB	20000	8055	14727
PUMSB	30000	8040	13741
PUMSB	50000	8160	13536

## Conclusion and future work

In this paper, the novel method for mining different association rules for the combination of low-frequency itemsets with the high frequency itemsets is proposed. Our approach uses different combination of high frequency itemsets (having low or high utility), with the low-frequency itemsets (having low or high utility). The combination of utility with frequency helps us to derive different association rules to increase either the utility, frequency, or both for the low-frequency itemsets in a transaction database. An extensive experiment on the different transaction databases, and the input data proves that these different association rules are important measure to decide different business strategies. Single phase FP-Growth [3] algorithm is used to generate candidate itemsets, calculate the frequency, utility, support, and confidence measure to generate the association rules. This approach generates different combination of the itemsets, and calculate all the required measures for generating association rules. Since the FP-Growth [3] is used to generate the candidate itemsets based on the support values, we use the index structure for the calculation of utility values. In future, the efficiency of the algorithm can be improved to calculate the utility itemsets. We intend to use the approaches descried in the advanced algorithms to generate the high utility itemsets without candidate generation, and thus reduce the time required to generate our desired association rules.

## Supporting information

S1 Transaction Datasets for Frequency and Utility Mining As mentioned in the experiment results section, we divide the data in small and large datasets. The small datasets for calculating the frequency of itemsets in transaction database contain *Accidents, Chess, Connection, Mushroom, PUSBM, and Retail* [32] transaction datasets. There are 500, 1000, 2000, and 5000 transactions per dataset. The small datasets for calculating the utility of itemsets in a transaction database contain *Accidents, Chess, Connection, Mushroom, PUSBM, and Retail* [32] transaction datasets. There are 500, 1000, 2000, and 5000 transactions per dataset. The large datasets for caluclating the frequency of itemsets in a transaction database contain *Accidents, Connection, and PUSBM* [32] datasets. There are 10000, 20000, 30000, and 50000 transactions per dataset. The large datasets for calculating the utility of itemsets in a transaction database contain *Accidents, Connection, and PUSBM* [32] transaction datasets. There are 10000, 20000, 30000, and 50000 transactions per dataset.
(ZIP)
